# Sustainable Exploitation of *Posidonia oceanica* Sea Balls (Egagropili): A Review

**DOI:** 10.3390/ijms24087301

**Published:** 2023-04-14

**Authors:** Odile Francesca Restaino, Concetta Valeria L. Giosafatto, Seyedeh Fatemeh Mirpoor, Marcella Cammarota, Sondos Hejazi, Loredana Mariniello, Chiara Schiraldi, Raffaele Porta

**Affiliations:** 1 Department of Chemical Sciences, University of Naples “Federico II”, Montesantangelo Campus, Via Cinthia 4, 80126 Naples, Italy; odilefrancesca.restaino@unina.it (O.F.R.); giosafat@unina.it (C.V.L.G.); seyedehfatemeh.mirpoor@unina.it (S.F.M.); sondosmohammadhasan.hejazi@unina.it (S.H.); loredana.mariniello@unina.it (L.M.); 2 Department of Experimental Medicine, University of Campania “Luigi Vanvitelli”, Via De Crecchio 7, 80138 Naples, Italy; marcella.cammarota@unicampania.it (M.C.); chiara.schiraldi@unicampania.it (C.S.)

**Keywords:** cellulose, egagropili, holocellulose, lignin, marine waste, *Posidonia oceanica*

## Abstract

*Posidonia oceanica* (L.) Delile is the main seagrass plant in the Mediterranean basin that forms huge underwater meadows. Its leaves, when decomposed, are transported to the coasts, where they create huge banquettes that protect the beaches from sea erosion. Its roots and rhizome fragments, instead, aggregate into fibrous sea balls, called egagropili, that are shaped and accumulated by the waves along the shoreline. Their presence on the beach is generally disliked by tourists, and, thus, local communities commonly treat them as waste to remove and discard. *Posidonia oceanica* egagropili might represent a vegetable lignocellulose biomass to be valorized as a renewable substrate to produce added value molecules in biotechnological processes, as bio-absorbents in environmental decontamination, to prepare new bioplastics and biocomposites, or as insulating and reinforcement materials for construction and building. In this review, the structural characteristics, and the biological role of *Posidonia oceanica* egagropili are described, as well as their applications in different fields as reported in scientific papers published in recent years.

## 1. Introduction

### 1.1. Biological Role of Posidonia oceanica and Derived Egagropili

**Figure 1:**
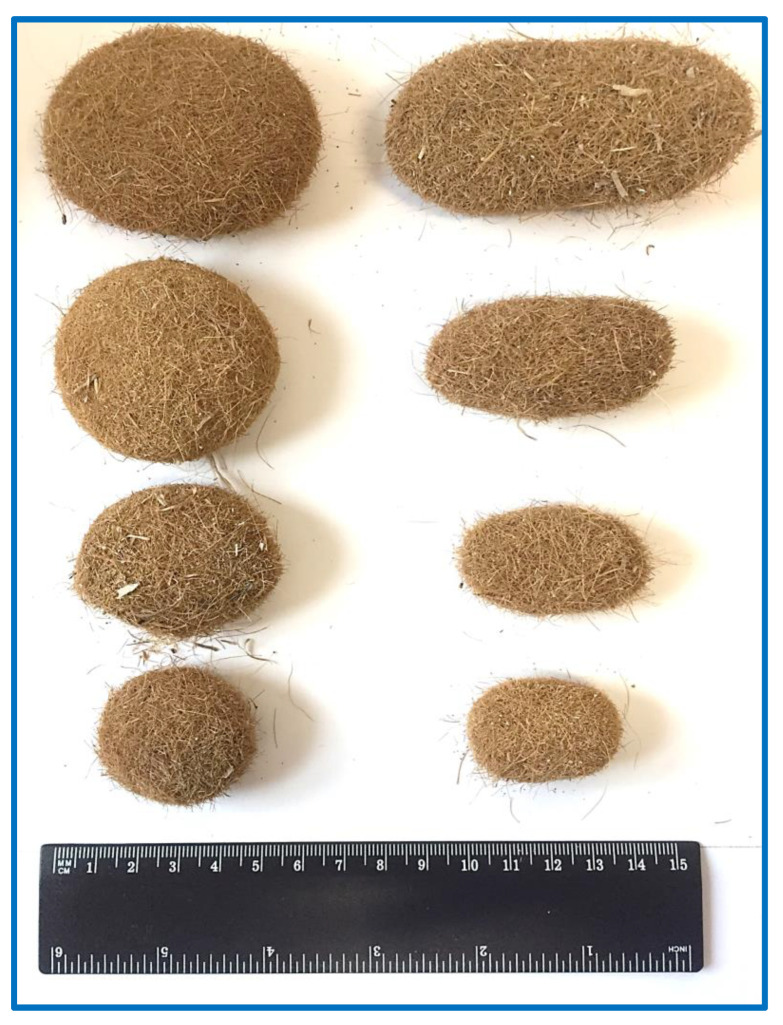
Pictures of POEGs of both oval and elongated shapes of different sizes, with a ruler used as a reference. They were collected by the authors on the beach of Marzamemi (Sicily, Italy; 36°44′34″ N, 15°7′1″ E).

**Figure 2:**
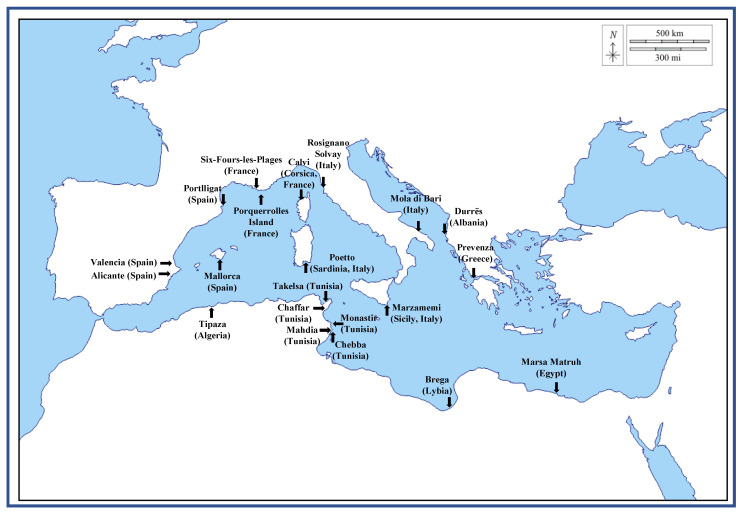
Map of the sites in the Mediterranean Sea where the POEG samples mentioned in this review were collected.

*Posidonia oceanica* (L.) Delile (PO) is an aquatic plant that is a dominant and endemic sea grass of the Mediterranean basin belonging to the *Posidoniaceas* family [1], and therefore, it is not an alga, despite still being frequently and wrongly defined as such in numerous scientific papers [1]. PO is a slow-growing plant that might live for millennia, and it forms huge underwater meadows that are estimated to cover more than 2.0% of the Mediterranean seabed (for a total of more than 12,000 km^2^) and that could extend from the sea surface up to 40 m of depth [1,2]. PO mainly spreads itself by sexual reproduction, although asexual reproduction might also occur by stem extension. The sexual annual reproductive cycle starts in the fall with pollination, and after a period of six to nine months, its mature fruits release seeds that reach the seabed and can develop roots and produce a new plant [3]. In only one year, the meadows of PO produce a huge amount of debris as leaves (leaf blades and sheaths), rhizomes, and roots that could constitute a biological sediment on the seabed and, eventually, might be either degraded by macro-organisms, microorganisms, and abiotic factors, or being subjected to the hydrodynamic action of the sea and rolled on its floor or ripple marks, or transported by the waves on the coasts where they accumulate [3]. The leaves, in particular the portions called leaf blades, fall from the plant after 5–8 months of existence and, generally, at higher rates in the autumn, as in this season, a lower amount of sunlight reaches the sea, and many strong storms occur. Conversely, the leaf sheaths usually remain attached to the rhizomes. The leaves, that reach the seashore transported by the waves and deposited on the beaches by the winds, usually form huge banquettes with thickness ranging from a few centimeters up to 2.5 m [3,4]. The annual total amount of PO leaves that reach the coast is in the range of 5 to 50 million tons, and nowadays the banquettes are estimated to cover about 50,000 km^2^ of sandy shores in the Mediterranean areas [1,2,3,5]. They are generally composed of wet and dried brown PO leaves, and they play an important ecological role in preserving the ecosystem and the biodiversity. In fact, they represent a favorable habitat for many species, promote sediment entrapment and stabilization, regulate the CO_2_ absorption of the sea and of the atmosphere, as well as water oxygenation, and protect the coasts from erosion by acting as a barrier [2,4,5,6]. PO roots and rhizome fragments, instead, might naturally be entangled by the constant rolling of the sea motions and aggregate as ball-shaped materials that are then delivered by the waves on the coasts, where they dried under the sun and the wind action [2,4]. These brown dried fibrous balls are generally known as PO egagropili (POEG), also spelled egagropilia, egagropoli, or aegagropiles, and reported as sea balls, sea rissoles, sea potatoes, beach balls, Neptune balls, or Kedron balls. The name “aegagropiles” derives from the ancient Greek words of αίγαγρoς (wild goat) and πῖλoς (fur), as the shape of these sea balls is reminiscent of the ones that are generally regurgitated by goats [4]. Figure 1 shows a re-presentative image of some POEG samples collected by the authors on the beach of Marzamemi, Sicily, Italy; 36°44′34″ N, 15°7′1″ E, and Figure 2 indicates the map of the sites in the Mediterranean Sea where the POEGs mentioned in this review were collected [2,5]. As with theleaves, every year millions of POEGs are delivered on the beaches by the winds, mainly in the period between October and March and especially after strong sea storms [3,5]. In some cases, POEG deposition could constitute a characteristic geomorphological feature of the landscape, such as along the southeastern Gulf of Sirte, in Libya, near the coastal town of Brega (30°26′06″ N, 19°40′01″ E) (Figure 2). Here the POEGs are deposited by the action of westerly winds, while the hot and arid wind of Ghibli, from the south, carries huge amounts of Sahara sand, thus forming peculiar POEG sandy sheets and dunes that are considered paleoenvironmentally interesting to study in our Holocene era [5]. Indeed, POEGs have been frequently studied in integrated archaeological and geological investigations as a sign of the coastal barrier evolution in different Mediterranean areas as well as of the stratification and of the climate changes during the Holocene (e.g., the studies on the Mistras coastal barrier system in central Sardinia, Italy [7]).

### 1.2. POEG Structural Characteristics

**Figure 3:**
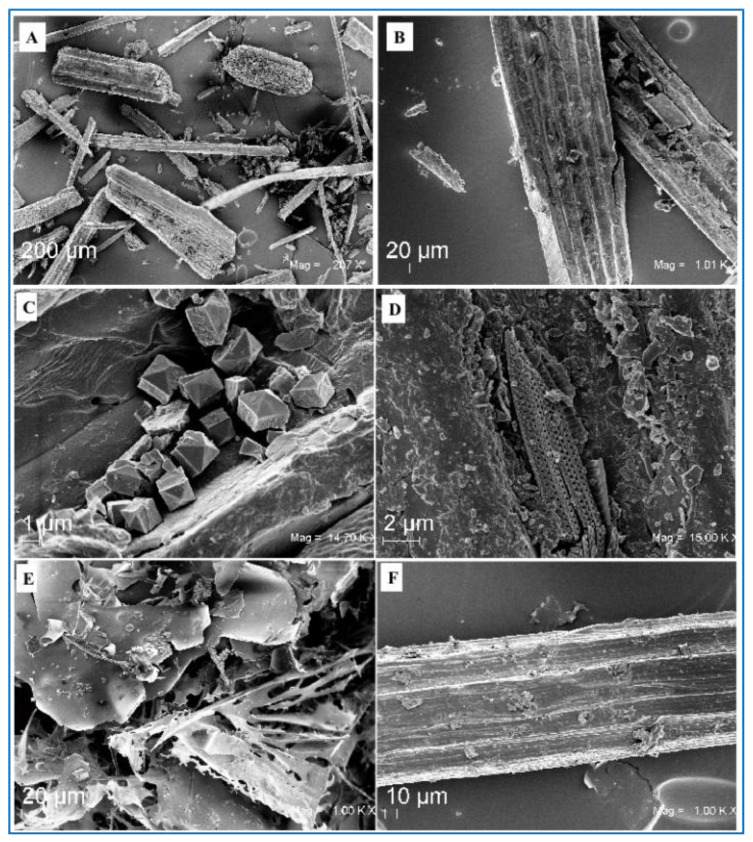
SEM pictures of the fibers of POEGs collected in Poetto Beach (Cagliari, Sardinia, Italy; 39°12′00″ N, 9°09′33″ E) (**A**) at low magnification (207X, scale bar 200 mm) and (**B**) at high magnification (1000X, scale bar 20 mm); (**C**) pictures of the crystals and (**D**) of the diatoms found on these POEG fibers (magnifications 14,7 KX and 15 KX, scale bar 1 and 2 mm), and (**E**) of the lignin and (**F**) of the holocellulose fractions extracted from them (magnification 10KX, scale bar 1 and 2 µm). [The preparation procedure of the samples for SEM analyses was similar to the one reported before (Restaino et al., 2022 [8]): Samples were suspended in 4% formalin in PBS for 18 h, dehydrated in increasing ethanol concentrations (from 30% to 100% for 5–15 min), dried in a critical point dryer, and sputtered with platinum-palladium (sputter coater Denton Vacuum Desk V). Fe-SEM Supra 40 Zeiss (5 kV, detector InLens) and Smart SEM Zeiss software were used for observation].

POEGs have specific physical characteristics such as a texture of rough felt, oval shapes (ball-shape), spherical or subspherical, or sometimes also elongated ellipsoidal shapes (egg-shape) and have different sizes with diameter values from millimeters to centimeters up to 20 cm (Figure 1). They have a very light weight, so they easily float freely in the sea water [2,3,5]. Their shape and their geometrical and mechanical properties are conserved, although they are formed in the open sea and under not-constant environmental conditions. For these reasons, POEGs have been considered a natural archetype of fiber networks and studied as models to understand fundamental aspects of the clustering mechanisms and of the aggregation dynamic forces in the networking processes [4]. Indeed, the natural processes and forces, as well as the sequence of events that drives POEG formation, are not easy to decipher, and in the literature, there are only a few studies on their origin, properties, and structural composition. In a recent paper, the determination of the average mass, average size, in terms of length and radius, and of the volume was performed on 2000 POEG samples collected in two beaches in France, at Six Fours (43°63′06″ N, 5°49′20″ E) and at Porquerrolles Island (43°00′02″ N, 6°13′38″ E) (Figure 2) [4]. X-ray tomography analyses were used to determine the POEG average density and density profile, their internal structure, and their fiber orientation. The sea balls showed an ave-rage density of 128 kg·m^−3^ and an inhomogeneous fiber alignment, at least in the dense outward shell, in which the fibers had low orthoradial orientation [4]. Studies on the fiber properties by using scanning electron microscopy (SEM) analyses were also performed to study the nature of the constituent fibers of the sea balls and thus decipher their connectivity and mechanical responses, which in general depend not only on the properties of the fibers themselves but also on their density and their organization in the cluster. Figure 3A, B report representative SEM images of the POEG fibers. Images of the outer and near-surface areas of the sea balls showed fibers to be very smooth and straightly slender with a cross-section of 100 µm with length values between 0.5 and 20 mm, and a predo-minance of short fibers with a weighted average length of 7.7 mm [4]. Analyses also showed that the connectivity and the cohesion mechanism between the fibers were due to their elasticity and inter-fiber friction contacts. The very dense structure and the high inhomogeneity of the fiber organization might be responsible for the high stiffness of POEGs that allows them to resist finger pressure. Based on all these data, the authors formulate the hypothesis that the sea balls are generally formed underwater, in submarine hollows, by isotropic mixing of randomly oriented smooth fibers and by aggregation mechanisms due to the action of friction forces from repeated collisions with the seabed and not by simple compaction processes due to hydrodynamic forces [4]. The hypothesis of POEG formation by aggregation mechanisms of litter fibers was also supported by the studies of Lefebvre and coworkers that collected 159 POEGs on the beach of Calvi (Corsica, France; 42°35′04″ N, 008°43′39″ E) (Figure 2). These authors performed deeper studies and formulated broader conclusions on the POEG formation process. In fact, the 159 samples were analyzed from a macroscopical point of view, in terms of shape, size, and density, but also in their microscopic aspects, performing sections of the balls by sli-cing them, studying both their external and internal layers, and carefully disassembling the POEG network to analyze the fibers at different strata [3]. Between the collected samples, only 21% had a ball-shaped form, while the rest showed an egg-shaped form. They had density values between 100 and 400 kg·m^−3^ with an average of 220 ± 40 kg·m^−3^ [3]. Thanks to stereo-microscope photographs and X-ray projections, the authors were able to classify the sea balls into three types: a heterogeneous type with a white, distinct nucleus that contains inside a piece of rhizome (58% of heterogeneous samples were egg-shaped, ellipsoidal POEGs), a homogeneous type without a distinct nucleus (mainly ball-shaped, spheroidal POEGs), in which the nucleus was probably degraded, and an intermediate type in which the nucleus contained clusters of dark pieces or fibers. Besides the nucleus (that, if present, might constitute 17% of the POEG architecture), all three types of POEGs showed three different concentric layers with diverse thickness and fiber composition: the superficial layer (14% of the POEGs), the median layer (25% of the POEGs), and the deep layer (44% of the POEGs). In the external, superficial layer, the fibers were poorly organized, as previously described by Verhillea and co-workers [4]. The median layer appeared fiber-rich, while in the deeper layer, many particles of sand contributed to a more whitish color. In fact, these round-shaped conglomerations contain not only fibers from the roots and rhizomes of PO meadows but also 10–20% of their weight as water and some minerals, such as aluminum-silicate particles and calcium carbonate salts, probably coming from the seabed sediments and included in the POEG network during their formation (Figure 3C) [3]. Sometimes calcified fragments of marine organisms, such as diatoms, might be easily detected in the POEG network too, not only by using X-rays but also by SEM analyses, as reported also by Restaino and co-workers [8] for samples collected in Poetto (Sardinia, Italy; 39°12′00″ N, 9°09′33″ E, Figure 2 and Figure 3D). Independently from the POEG type, the nucleus and the three layers always showed thickness values that increased gradually from the external portions to the internal parts, ranging from 2.35 to 7.95 mm. The fiber density and the presence of mineral particles also increase when mo-ving from the external to the internal part of the POEG network. Fibers ranged from 12.0 ± 2.0% of the section surface of fiber/unit of section surface in the superficial zone to 18.0 ± 4.0% in the median and deeper layers, while minerals ranged from 1.2 ± 0.9% of the section surface of mineral/unit of section surface to 7.0 ± 2.0%. In a heterogeneous POEG, the nucleus is the denser part (about 26.0 ± 4.0%), and it sometimes also contains woody pieces and minerals. This central zone, if present, also contains larger fibers. In fact, the determination of the width of 960 fibers in the three layers and of the nucleus of the different types of POEGs allowed the authors to classify them into thin, flat, and wide fibers. The thin fibers (0.142 ± 0.051 mm) were mainly present in the superficial layer (70% and 55% of the superficial layer fibers were thin in the homogenous and heterogeneous types, respectively), similarly to what had previously been reported by Verhillea and co-workers [4]. In the median layer, a mixture of tin, flat (0.462 ± 0.095 mm), and wide (2.295 ± 1.380 mm) fibers was detected. In the deep layer, a higher percentage of wider fibers was detected in heterogeneous POEGs (24%), compared to the homogeneous type (5%) [3]. All the fiber types were flexible, especially the thinner ones. Based on all these results, the authors proposed that the formation of the POEGs follows several steps in which an initial roll of fibers and sand starts to aggregate in the seabed on ripple marks, then this roll grows and breaks down into small balls by mechanical and/or microbial degradation [3]. Structural characterization of the POEG fibers showed a chemical composition typical of the lignocellulosic materials, thus made of lignin groups bound to a holocellulose structure that includes cellulose and hemicellulose chains (Figure 3E,F) [2]. The composition of the POEGs was first reported as being made of 29.8% lignin, 61.8% holocellulose (21.8% hemicellulose, and 40% cellulose), and then ashes [6]. But then more accurate analyses were performed; acidic hydrolysis and HPAE-PAD analyses were used to determine the monosaccharide composition of untreated POEG fiber samples collected in Poetto. They were neutral sugars, such as fucose (Fuc), arabinose (Arab), rhamnose (Rham), galactose (Gal), glucose (Glc), and xylose (Xyl), as well as an amino sugar, such as glucosamine (GlcN), and an uronic acid, such as GlcA. The representativity of these sugars ranged from 51.0% of Glc to 0.6% of Fuc, for a total carbohydrate content of 28.3% of the dry weight of the fibers. Monosaccharides such as Fuc, Arab, Rham, Gal, Glc, Xyl, and GlcA were also found in the water extracts of PO leaves, together with mannose (Man) and galacturonic acid (GalA), but with a relative abundance that ranged from 80% for Gal to 50% and 45% for Glc and Xyl, respectively [9]. In another paper, aqueous fractions were extracted from samples of POEG, collected in Tunisia (the specific place was not mentioned), and then analyzed by GC-FID/MS and by colorimetric assay for the monosaccharide composition, by the permethylation method for the identification of glycosidic linkages, and by elemental analyses [10]. These authors found the same monosaccharide composition described in the previous paper (Fuc, Arab, Rham, Gal, Glc, Xyl) and detected the presence of uronic acid by colorimetric assay, but they found that xylose was the most representative sugar, which may be because they analyzed the water extracts of POEGs and not the fibers directly [10]. Linkages of 1,2-, 1,3, 1,4-types were found by the permethylation method for the Arab, Rham, and Xyl monosaccharides, but the complete structure of the carbohydrate sequence was not determined. Both cellulose and hemicellulose fractions were isolated from POEG fibers mainly by u-sing protocols that first removed the lignin portion. In the literature, different methods have been used to isolate the lignin-containing fraction from POEGs after an initial washing step, and the samples were processed through grinding or knife milling. In the first protocol, the powder was extensively extracted by using the Soxhlet method and then with a dioxane–water solution to remove plant cell-wall components and recover a total content of acid-soluble and acid-insoluble lignin [11]. In the second protocol, instead, the powder was treated using a 1.7% sodium chloride oxidation at pH 3.5 and then dewaxed by employing a toluene/ethanol solution (2:1 *v*/*v*) [2]. The total lignin content was estimated to be around 8–12% of the initial weight in the POEG samples collected in Portlligat Bay (Girona, Spain; 42°17′37″ N, 3°17′7.2″ E, Figure 2) that were extracted with the first protocol [11], or about 15% of the initial weight in the case of POEG samples collected in Poetto Beach (Sardinia, Italy; 39°12′00″ N, 9°09′33″ E, Figure 2) and extracted with the se-cond method [2,12] (Figure 3E). The analyses of the lignin of the egagropili collected in Portlligat, performed through derivatization that was followed by the reductive cleavage (DFRC) degradation method, and by GC/MS analyses after pyrolysis and by bidimensional NMR analyses, showed the presence of few syringyl units and numerous guaiacyl units that were also frequently bounded to a highly acetylated 4-hydroxybenzoic acid [11]. The presence of a high degree of p-hydroxy benzoylation in both guaiacyl (73%) and syringyl (61%) lignin units in *P. oceanica* tissues was the highest ever reported, so far, in a plant, and explained the higher capacity of this species to store carbon dioxide, compared to other species, such as *Posidonia australis* [13]. Furthermore, gel permeation chromatography analyses determined the molecular weight of this lignin fraction that was found to be 6100 Da, with a polydisperdity of 2.2, thus indicating a low molecular weight aromatic polymeric structure, made of dimers, trimers, or tetramers. Instead, by using the second protocol of extraction, a water soluble, brown colored lignin-containing fraction was isolated that showed high absorbance in wavelength range from 250 to 400 nm, due to the presence of aromatic rings. This presence was also confirmed by broad peaks in the aromatic regions (6.5–7.5 ppm) in ^1^H NMR analyses [12]. In the alkoxide regions (3.5–4.5 ppm), the signals of -OCH_3_ groups indicated the presence of the syringyl and guaiacyl units. After determination of the total phenol content by the Folin–Ciocalteu method, 22% of flavonoids and 10% of anthocyanins were identified using an aluminum chloride co-lorimetric assay [12]. The sodium chloride oxidation method allowed the extraction of the part of the hemicellulose chains that remained attached to the lignin portion, as demonstrated by the presence of alkoxy signals in the ^1^H NMR spectrum. This lignin–carbohydrate complex showed a molecular weight (Mw) of about 35 kDa and a monosaccharide composition made of the eight different sugars (fucose, arabinose, rhamnose, galactose, glucose, xylose, glucosamine, and glucuronic acid) that was found in the POEG fiber mo-nosaccharide composition as well, but, of course, with different ratios as the glucose pre-sence due to the cellulose chain, was greatly reduced by the carbohydrate extraction [12].

## 2. *Posidonia oceanica* Egagropili as Lignocellulosic Biomass to Valorize

**Figure 4:**
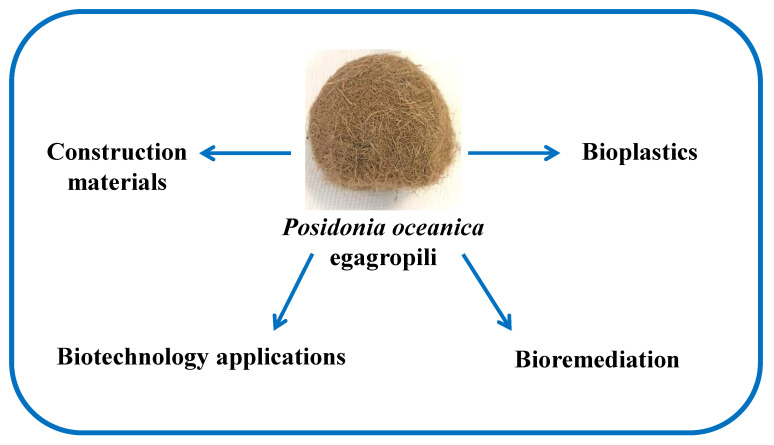
Fields of applications of POEGs.

Every year, huge quantities of POEG fragments accumulate on Mediterranean coasts, resulting in a problem and a negative visual impact, creating the necessity for municipa-lities to remove this waste to keep beaches cleaned and ready for tourists and the summer season [6]. As disposal of this waste is performed every year and does not have negligible costs, it would be useful to find a way to valorize POEGs as readily available, low-cost, and renewable lignocellulose biomass and as a good source of cheap materials and fibers to produce added-value products from the perspective of an eco-friendly society [1,10,14]. The whole POEGs, themselves, have interesting properties that can be used for energy production [15], dye removal from the environment, building materials in the construction sector, as well as in the development of new packaging systems. They are also an optimal source of lignin and cellulose fractions that might be widely used in the paper-making industry, as well as a source of carboxymethyl cellulose in the production of fiber-reinforced composite materials and biopolymeric films. Lastly, they might be used as carbohydrate substrates to grow microorganisms in biotechnological processes. The lignin fraction, made of numerous 4-hydroxybenzoic acid groups, might instead be used for the synthesis of chemicals, such as parabens, and pharmaceutical molecules, such as paracetamol. All new applications of POEGs and their derived fractions are reported in the following paragraphs from the literature published in the last few years in the fields of biotechnology, environmental decontamination and bioremediation processes, bioplastic and biocomposite preparations, and construction materials (Figure 4 and Table 1).

### 2.1. Posidonia oceanica Egagropili in Biotechnological Applications

The whole POEGs and/or their lignin and cellulose fractions have been employed in numerous biotechnological applications (Table 1). For example, they have been recently used as substrates for microorganism growth. Recently, POEGs collected in Poetto (Figure 2) were added, after being washed and milled, as a raw source, in a glucose, yeast, and malt extract containing medium to boost the natural synthesis of melanin by *Streptomyces roseochromogenes* [8]. The researchers found that the addition of 2.5 g/L of POEG powder in shake flasks enhanced the biomass formation of 1.5–1.9 folds in a 120-hour run at 26 °C and 250 rpm and increased the melanin production of 7.4 times, up to about 3.9 g/L, compared to the control. In 2-liter batch experiments, the melanin production reached values of 9.2 g/L in only 96 h, with a further increase in both production and productivity of about 2.4 folds [8]. Melanin is a secondary metabolite that starts to be synthesized by streptomycetes in their late exponential and stationary phases of growth. The first two reactions in the melanin synthetic pathway are catalyzed by the action of a tyrosinase that converts the precursor L-tyrosine to L-3,4-dihydroxyphenylalanine (DOPA) in the presence of molecular oxygen. The scientists discovered that the addition of the POEG powder to the medium enhanced the tyrosinase activity of 1.5–1.7 folds compared to the control during the microorganism growth and drove to a higher and faster melanin production. Furthermore, studies have demonstrated that both the lignin and cellulose components of the POEGs are necessary to boost melanin production, but in different ways. The holocellulose chains were used by the bacteria as a substrate for their growth, whereas the isolated lignin carbohydrate complex was necessary to induce melanin synthesis, but only if the two components were supplied together higher biomass values and melanin concentrations were obtained [8]. In another paper, instead, only the cellulose component, isolated from the POEGs collected in Poetto again, was used to enhance the production of cellulases in the same strain, *S. roseochromogenes* [16]. This fraction, supplemented to the growth medium at a concentration of 2.5 g/L, was able to increase the enzyme production in shake flasks of 4.3 fold compared to the control, aup to 268 U/L in 72 h with a productivity of 3.7 U/L/h and up to 347 U/L in 45 h in 2-liter batch experiments with a productivity of 7.7 U/L/h. The supplementation of cellulose induced the expression of a pool of three cellulases with molecular weights of about 115, 63, and 47 kDa. This pool had optimal activity at 60 °C and pH 5.0, and showed their ability to hydrolyze at the same time substrates such as carboxymethyl cellulose (CMCase activity) and filter paper (FPase activity), and it also showed a β-glucosidase action [16]. As POEGs contain salts in their fibers, they might also constitute an example of marine-origin lignocellulose biomass, a waste that needs to be studied to improve our knowledge of the bioconversion of sea raw materials into fermen-table sugars to produce second-generation biofuels [17]. In a recent paper, the POEGs collected on the beach of Takelsa, Tunisia (36°47′24″ N, 10°37′48″ E) (Figure 2) were used as substrates to check the capacity of cellulose/hemicellulose degradation enzymes expressed by the plant growth-promoting actinobacterium, *Microbacterium metallidurans* TL13. Genomic studies on this strain have shown that it has genes decoding for carbohydrate active enzymes (CAZymes), such as CMCases, β-glucosidases, and xylanases. The POEG fibers were first pretreated by an autoclave at 120 °C for 20 min and then used in enzymatic assays with *Microbacterium metallidurans* TL13; enzyme preparations (20 FPase/g of dry substrate) by incubation at 180 rpm and 37 °C at 180 rpm were conducted for 72 h. The microbial enzymes were extremely halostable and osmoadaptive, and they were able to efficiently degrade POEGs by converting it into fermentable sugars with a yield of about 33% of the theoretical maximum yield, thus showing a promising, green biocatalytic tool for seagrass-based biorefinery [17]. In the literature, not only it was reported the enzyme-assisted hydrolysis of POEGs—but also thermomechanical—and chemical-based methods. A combination of both these methods was used by Kadraoui and co-workers [18], who set up a new pre-treatment process for POEGs with the aim of producing nanofibrillated cellulose to be employed in efficient bioconversion processes. Fibers of sea balls collected on the beach of Monastir, Tunisia (35°46′40″ N, 10°49′34″ E) (Figure 2) were first delignified by using 20% sodium hydroxide and 0.1% anthraquinone and then bleached with sodium chlorite. The obtained pulp was dried, milled, and then subjected to a TEMPO (2,2,6,6-tetramethylpiperidine-1-oxide radical)-mediated oxidation (TEMPO/NaBr/NaClO 0.1/1/6.22 at 25 °C) to convert primary hydroxyl groups to carbo-xylate functions. Then their pulp was exposed to steam explosions at 170 °C, at 8 bar for 30 s to obtain microfibrillated cellulose [18]. The POEGs were also used in innovative and ecofriendly fortification approaches, such as improving edible plant growth. In the literature, a recent study reported that POEGs collected on the beach of Mola di Bari in Italy (41°3′36″ N, 17°5′24″ E) (Figure 2) were used as supplements for the medium composition of two different brassica microgreens (Mizuna and Rapini) [19]. The POEG fibers were mixed with a commercial medium at rates of 25, 50, and 75% to constitute a natural organic matrix to improve the nutritional quality of the two brassica plants. This supplementation was effective in improving the iodine (I) and boron (B) contents of the microgreens up to 50 and 75%, and thus their nutritional profiles [19]. POEG balls collected on Oliva and Denia beaches in Valencia, Spain (39°28′11″ N, 0°22′38″ W) (Figure 2) were instead used as a medium to improve the germination rate and average germination time of lentils compared to those seeded on cotton [20]. The germination rate was reduced of around 17% on washed and milled short fibers (2–5 mm) and of about 42% on bleached, original long fibers (6–15 mm), while the average germination time was reduced up to 2.8 days.

### 2.2. Posidonia oceanica Egagropili in Environmental Decontamination Processes

PO is a natural biomonitor of pollution in the sea as it catches heavy metal ions such as arsenic (As), cadmium (Cd), lead (Pb), and zinc (Zn) present in the water to store them in its organs at concentrations of up to several mg/kg [21]. Additionally, the dried fibrous balls of POEGs have promising features, such as being green adsorbents for cationic pollutants since they are already found in nature as cation-imprinted lignocellulose networks that contain a high presence of salts [21] (Table 1). The high biosorption properties of POEGs could constitute a new potential way for their valorization and reusing in cleaning systems of blackish water decontamination processes from dyes, phenol compounds, and heavy metals [10,21]. According to some authors, the POEGs’ ability to uptake metals might be due to the porosity of their cell walls, which allow the entrance of small ions, and due to their lignocellulose composition [22]. In the literature, many studies have focused on POEGs’ absorption abilities and their possible applications in diverse deconta-mination processes. In these investigations, some key parameters of the process have been taken into account, such as the pH of the contaminant solution, the temperature at which the process is performed, the contact time between the solution and the POEGs, the initial concentration of the contaminant, the amount and size of the POEGs, and the enthalpy and entropy of the process. In a recent paper, some POEGs collected in Marsa Matrouh, Egypt (31°21′15″ N, 27°14′14″ E) (Figure 2) were “activated” by soaking them after pulve-rization in 1 M acetic acid overnight and then used as an eco-adsorbent for the removal (in 30 min of shaken run) of methylene blue and lead ions (Pb^2+^) present in aqueous solutions in concentration ranges from 0.6 to about 2.6 g/L [21]. The initial acetic acid activation method resulted in greatly reducing the presence of some ions, such as barium (B), cadmium (Cd), chromium (Cr), copper (Cu), magnesium (Mg), and zinc (Zn), in the fibers that are naturally present in the collected samples without altering the lignocellulose composition. On the basis of these analyses of the adsorption isotherms and thermodynamic studies, the authors proposed that the POEG adsorption mechanism of methylene blue, present in water as monomers, dimers, or trimers, might be due to combined electrostatic and physical multi-layer adsorption processes, whereas the lead was chemically adsorbed. The activated fibers were then applied to decontamination of waste blac-kish waters to remove the methylene blue from Manzala Lake, Egypt, with an efficiency of 91.5–99.9% [21]. The use of POEGs as new biosorbents for heavy metals (M) with an oxidation state of II was investigated in another paper using samples collected in Tipaza, Algeria (36°37′4″ N, 2°23′28″ E) (Figure 2). The balls were washed, dried at 40 °C for 48 h, and ground to obtain a size fraction lower than 160 µm. Then, they were employed in batch experiments in water solutions with metal ions of Pb^2+^, Cu^2+^, Ni^2+^, Zn^2+^, and Cd^2+^ [22]. Physical parameters that influenced biosorption were investigated, such as the pH, the temperature, the metal concentrations, and the contact time with the POEG fibers. According to these authors, the cylindrical shape of the POEG fibers plays an important role in the adsorption and precipitation of the metal ions on the external surface. Maximum absorption was reached in 80 min at a pH range of 6.0–8.0. Basic pH values made the process quicker, as the metal ions more easily interacted with the negatively charged surface made by the carboxyl, hydroxyl, and amine groups of the lignocellulose structure. Higher absorption was also achieved at 40 °C, which is a temperature value that helped the coordination process of the metal ions with the homogeneous surface binding sites of the fibers. The process of chelation resulted in spontaneous behavior, although positive enthalpy data suggested that the phenomenon was endothermic [22]. Pb^2+^, Cu^2+^, Ni^2+^, Zn^2+^, and Cd^2+^ were adsorbed up to maximum concentrations of about 48, 44, 41, 38, and 30 mg/g of POEGs, respectively, and the removal capacity was in a range from 70% to 98% [22]. In another paper, POEGs collected on the Monolithi beach of Preveza, in Greece (38°57′3″ N, 20°45′6.1″ E) (Figure 2) were employed for the removal of Cr^6+^ from wastewater in batch experiments after chemical activation [23]. The activated POEGs, obtained this time by treatment with KOH aqueous solution, showed a high surface area of up to 1563 m^2^/g, a cumulative pore volume of 0.74 cm^3^/g, and a maximum adsorption capacity of 120 mg/g for the fibers. The POEG adsorption reaction was endothermic, mainly due to electrostatic attractions of Cr^6+^ ions with the positively charged pores of the activated surface or by adsorption and complexion with oxygen-containing groups. In another recent paper, POEG samples collected on the beach of Chaffar, in Tunisia (34°33′50″ N, 10°33′50″ E) (Figure 2), were tested as possible pollution biosorbents to remove hydrocarbon spills from seas [24]. The authors tested them as intact raw materials or as milled fibers for their capacity to absorb pure oil, pure water and a mixed oil/water system in batch experiments. Moreover, in this paper, factors such as the oil concentration, the time of sorption, and the amount of sorbent were investigated to optimize this process. Raw and milled fibers were able to absorb 5.5 g/g and 14 g/g of pure oil and about 15 g/g and 16 g/g of water, respectively, whereas in the mixed system, a maximum oil and water sorption capacity of 4.7 g/g, 12.8 g/g, 7.4 g/g, and 8.3 g/g was found. The highest oil removal efficiency was obtained by using milled fibers with sizes in the range of 0.5–1.0 mm (length), which produced a maximum adsorption in 15 min. The isotherm curve analyses confirmed that, in this case, the oil absorption seemed more like a monolayer coverage process of homogeneous sites than a multilayer coverage process of a heterogeneous-like surface [24]. POEGs had significantly higher abilities for oil absorption capacities than other waste vegetable biomasses used as natural sorbents, such as bagasse, wheat, and barley straws, but lower abilities than those of cotton fibers or silk-floss fibers. POEGs collected on the Chebba beach, in Tunisia (35°14′24″ N, 11°07′12″ E) (Figure 2) were employed instead for the removal of antibiotics, such as oxytetracycline, that are present as micro-contaminants in water environments [25]. The POEGs were first washed with water to remove sand and salt residues, and then they were washed with ethanol. They were then dried and milled into particles of three different homogeneous sizes (100, 75, and 50 µm) before being used. The 50 µm size particle resulted in more efficient adsorption of the antibiotic for up to a maximum of 86%, starting with 80 mg/L as the initial concentration of the drug (when the process was performed at 25 °C with 20 min of contact). The pH value at which the experiments were performed was a key factor in the process because oxytetracycline has three pKa values (3.6, 7.5, and 9.4). The maximum adsorption was reached at pH 6.0, probably when hydrogen bond interactions were formed between the hydroxyl and phenolic groups of the lignocellulose surface of the fibers and the antibiotic. The adsorption process of the antibiotic was a spontaneous, exothermic, and entropically favorable process [25]. Whole POEGs are also a common fiber trap that involuntarily work in the sea to catch plastic wastes in the water [26]. Some researchers have estimated that they annually collect nearly 900 million plastic pieces and that around 1500 pieces of plastic are transported for every kilogram of POEGs. For example, it was found that 17% of 1 kg of POEG samples collected on the beaches of Mallorca Island, Spain (39°42′37″ N; 2°59′42″ E) (Figure 2) contained plastics of different sizes: 45.5% of the plastic pieces were microplastics (pieces smaller than 5 mm), 45.5% were mesoplastics (5–25 mm), and about 9.1% were macroplastics (higher than 25 mm). They ranged in size from 1.0 to about 59.0 mm, with an average length of 9.5 mm. Spectrometry analyses determined that they were mainly made of polymers, such as polyamide (10.8%), polyethylene (21.6%), polyethylene terephthalate (35.1%), polypropylene (13.5%), but also polystyrene, polyurethane, and polyvinyl chloride. These plastic debris were found to be in various forms, such as filaments and fibers (64.9%), fragments (21.6%), films (8.1%), and foams (5.4%). Their total weight was up to 13 mg for each POEG, a weight that constitutes around 0.15% of the POEG’s weight [26]. Considering all these data, POEGs might provide a valuable potential trapping service because they are able to sieve up to 867 million of plastic debris each year, which might be particularly important in the future for finding applications to preserve the ecosystem in the Mediterranean Sea, where a very high level of microplastic contamination persists both at the surface waters and seafloor.

### 2.3. Posidonia oceanica Egagropili in Bioplastic and Biocomposite Preparations

POEG fibers have been widely used to strengthen the matrix of plastics, both of bio-based and oil-based origins (Table 1). Mirpoor et al. [2] have exploited both the lignin-carbohydrate (LCC) and the nanocrystalline cellulose (NC) fractions, after extraction from egagropili collected in Poetto (Figure 2), as reinforcing agents for hydroplastic materials. The two fractions were able to improve the physicochemical properties of biodegradable films obtained from hemp (*Cannabis sativa*) oil seedcake protein concentrates. In fact, such materials exhibited a high tensile strength and Young’s modulus; the Young’s modulus increased from around 20 to 45 and to 80 MPa, while the elongation at break was reduced from 300% to 250% and to 120% in the presence of LCC and NC, respectively. They possessed barrier properties towards water vapor, O_2_, and CO_2_. In addition, both fractions decreased film hydrophilicity, infact, moisture content, solubility, and swelling ratio were lower for the films prepared in the presence of additives. In 2021, the same authors [12] investigated deeply the LCC fraction obtained from the same POEG samples from a chemical point of view using FT-IR and NMR analyses (see also Section 1.2). The LCC fraction was water soluble as it contained monosaccharides and exhibited a brownish-to-black color due to certain functional groups, such as phenylpropane-based polymers. Furthermore, it exhibited a remarkable and stable antioxidant activity that was easily released over 6 months when it was used as an additive in hemp protein-based films. On the other hand, in another paper, lignin-containing cellulose micro/nanofibrils (LCM/NF) were also obtained by combining the steam explosion process or twin-screw extrusion (as energy-efficient pretreatments) with a conventional grinding step [27]. The chemical composition of the fibers, collected in Monastir (Figure 2), before and after pulping, was analyzed. The obtained LCM/NF suspensions were characterized by several techniques, such as morphological and mechanical analysis. It has been shown that if the sulfonation method was coupled with steam explosion or twin-screw extrusion, then it was possible to obtain LCM/NF gels with relatively low viscosity and nano papers with a Young’s modulus of around 5 GPa. Sulfonation was revealed to be an effective pretreatment to lower the energy during grinding, and therefore it can be considered a valid technique to be applied in the field of packaging [27]. POEG fibers, collected in Campello Beach in Alicante, Spain (38°21′00.01″ N, 0°29′00″ W) (Figure 2), were exploited in the reinforcement phase and in oil-based polymeric matrices, such as the high-density polyethylene (HDPE) [28] samples), and more recently even in polyesters [28,29]. In the first work, the authors investigated the influence of POEG fibers and deinking paper sludge (DPS) on the thermo-mechanical properties of high-density polyethylene binary and hybrid composites [29]. POEG fibers (samples collected in Monastir, Tunisia) (Figure 2) were simply obtained by drying the washed balls at 80 °C in a hot air oven for 7 days and then reducing them to a powder by milling them with a grinder. They were subsequently sieved to an approx. 1 mm size. It was found that the properties of binary and hybrid composites depended on the chemical composition of the fillers, and the material’s mechanical properties depended on the filler content as well (POEG and DPS). A better interfacial adhesion between fillers and matrix was achieved in the presence of maleated polyethylene (MAPE). HDPE/POEG/MAPE composites achieved the highest tensile modulus and strength with 40% of the POEG fibers [29]. The effect of the POEG fibers on the mechanical properties and water absorption behavior of unsaturated polyester resin matrices obtained by the compression molding process was also evaluated in the following paper [30] (samples collected in Mahdia, Tunisia, 35°30′24″ N, 11°2′48″ E, Figure 2). The obtained results showed that the stiffness, strength, and hardness of the unsaturated polyester resin/POEG fiber composite increased with increasing concentration of fibers, demonstrating that the latter acted as matrix reinforcing agents. Different amounts (10, 20, and 30 %wt) of fibers from POEG collected on the beach at Rosignano Solvay, Livorno, Italy (43°19′1.7″ N, 10°27′52.7″ E; Figure 2) were obtained as described above and incorporated into a thermoplastic matrix made of poly(hydroxybutyrate-co-hydroxyvalerate) (PHBV). Moreover, acetyl tributyl citrate was used as a plasticizer to produce melt-processable and biodegradable composites for specialized applications in marine environments [31]. The produced materials were characterized in terms of processability, thermal/mechanical/morphological properties, and biodegradability under simulated and real marine environmental conditions. The obtained composites showed good thermal stability and mechanical properties, especially in terms of stress at break and impact resistance. The biodegradation test results in the simulated marine environment showed that the presence of POEG fibers in the composites accelerated the biodegradation of the polymeric matrix. Under real marine conditions, the samples with POEGs showed higher weight losses and mechanical feature reduction compared to those prepared without fibers. Due to the obtained results, the authors speculated that these biocomposites could be exploited as starting materials for low-cost and biodegradable items that are usable in the sea and/or sand dunes, increasing the market opportunities for biopolymers, such as PHBV, and at the same time, finding an eco-sustainable valorization for POEG residues accumulated in large quantities on Mediterranean shores [31]. More recently [32], the PHBV mixture with egagropili fibers was further employed to prepare novel bio-containers with the aim of restoring seagrass meadows and coastal dunes. The authors have discovered that the presence of such bio-containers on the coast has favored the establishment and spread of plants because they promoted shoot production, minimized transplant shock, and enhanced the capacity of plants to resist physical disturbances, such as those due to storm events. This novel approach is very useful for substituting polluting, non-biodegradable materials in recipient habitats [32].

### 2.4. Posidonia oceanica Egagropili in Construction Materials and as Decoration

**Figure 5:**
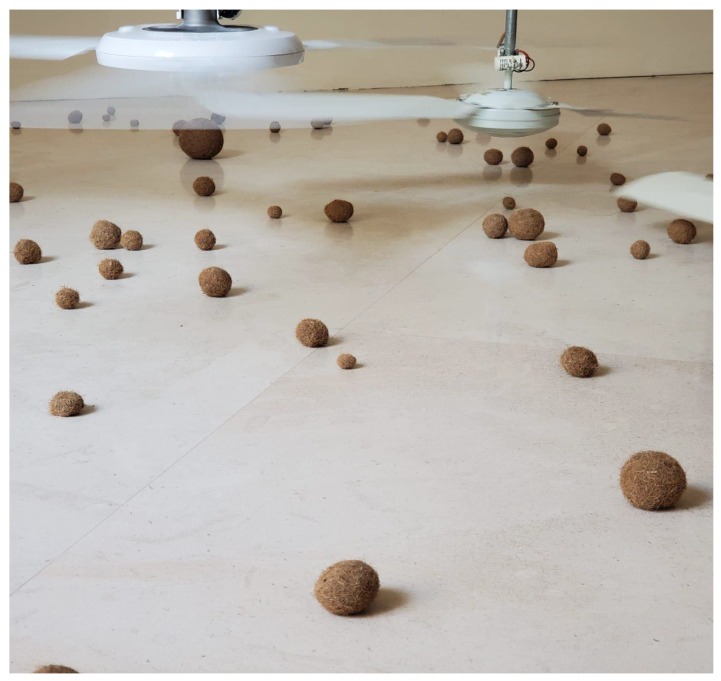
**Bruna Esposito** (Rome, 1960. The artist lives and works in Rome). **Venti di rivolta o rivolta dei venti** 2009. Fans, galvanized iron pipes, an electrical system, sea straw balls, and a breeze. (Reproduced with permission from the artist and by courtesy of Studio Stefania Miscetti-associazione culturale Mantellate).

POEGs have been widely studied in recent years as environmentally friendly materials to be employed in buildings having fire, sound, and water-resistant properties (Table 1). For example, POEGs have been used in the construction sector as insulating material to reduce the risk of energy source shortages and better manage energy consumption in buildings [33]. In this context, the use of insulating materials is a key factor, as they might provide better thermal comfort, sound insulation, and fire protection [37]. It is worth noting that so far, many of the insulation materials used in the building sector were petroleum derivatives, and they must be replaced with materials derived from renewable na-tural resources from the perspective of a sustainable bioeconomy [34]. Benjeddou et al. [35] studied the effect of adding POEGs to cement composites and found that the mecha-nical strength, sound, and thermal diffusivity were all improved. The POEG fibers were able to reduce sound transmission by increasing the fiber volume and, consequently, the air voids in the cement paste. Another study carried out by Jedidi and Abroug [34] reported that the addition of POEG fibers (collected in Monastir, Tunisia; Figure 2), up to 10%, to the plasters significantly improved their mechanical properties. Thermal conductivity also decreased from 0.35 W/m/K in the absence of fibers to 0.11 W/m/K in the presence of 20% of them. The physical-mechanical properties and the resistance to water and fire of the cement made with POEG fibers and the cement based on pine wood have been studied by Mayer et al. [36] (POEG samples collected in Durrës, Albania, 41°19′27″ N, 19°27′21″ E). The results obtained showed that the POEG fiber-based cement had higher mechanical strength and toughness compared to the pine wood-based cement due to its higher compatibility. In addition, the POEG cement showed a more homogeneous structure with better properties in terms of both water resistance and flame retardance. It has also to be mentioned that the POEGs are nowadays commercialized on the market under the names “*Posidonia* spheroids, Neptune grass, sea weed balls, or Mediterranean tape weeds” as marine-origin artistic materials to be used as decoration of houses and gardens or for boat garland, for handcraft works as insulating materials for apartment walls or roofs, and sold on-line in sets of 5 to 20 pieces at a price ranging from 6 to 25 euros for each set (see for example, https://www.etsy.com/, accessed on 12 April 2023). As decorative objects, they have also been used for artistic purposes by contemporary artists, as in the case of the masterpiece of Bruna Esposito (Figure 5).

## 3. Conclusions

In recent years, POEGs have caught more and more attention as interesting marine-origin raw materials. Due to their structural and physical properties, they have demonstrated that they could be employed in a variety of applications and fields. The whole POEG fiber network constitutes an interesting nutrient that can be supplemented to a medium for both plant and microorganism growth, while their cellulose components have already been used in biotechnological processes to obtain added valuable molecules, such as enzymes and biofuels. Due to their physical and mechanical properties, these fibers could easily constitute the base of newly developed bioplastics and biocomposites, as well as insulating materials for buildings. Moreover, their isolated lignin fractions might be easily used as reinforcement in newly designed biomaterials. The ability of their fibers to adsorb metals and water contaminants also makes POEGs the ideal ecological tools for environmental decontamination.

## 4. Future Directions

**Figure 6:**
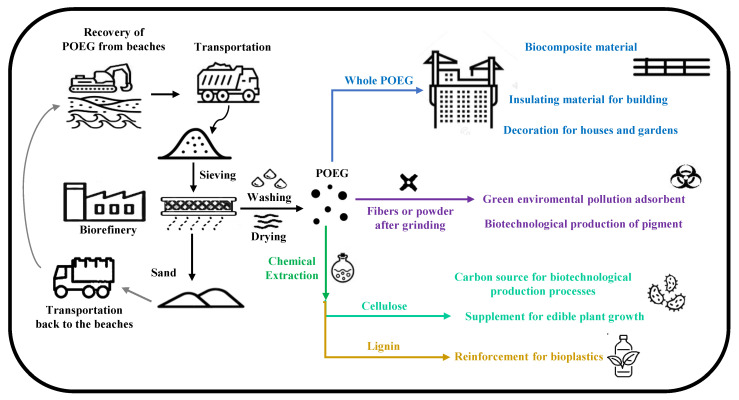
Steps of POEG recovery, transportation, and pre-treatment in a biorefinery (black arrows) before being used in several applications and fields, such as whole POEG (blue arrow); as fibers or powder after grinding (violet arrow); or after chemical extraction (green arrow) of its cellulose (light green arrow) and lignin fractions (yellow arrow). The recovered sand is transported back to the beaches (grey arrow).

POEGs have many useful structural, mechanical, and physical properties that make them useful in different applications and fields. Further increasing the possible and potential uses of POEGs might also help in the future to sustain the economic growth of all the countries that overlook the Mediterranean basin. As a matter of fact, as the PO is an endemic sea grass of the Mediterranean Sea, it could constitute an easy, low-cost, and natural source of POEGs that could be used as raw materials in different industrial processes and sectors, thus increasing the economy of the Mediterranean basin countries. In this perspective, on the basis of the data reported in the literature and mentioned here in this review, we imagined and designed a generic scheme of a hypothetical bio-refinery as a model for a start-up company that would eventually valorize the POEGs, in a perspective of more sustainable industrial processes and of a circular bio-economy (Figure 6).

## Figures and Tables

**Table 1:** Fields and types of applications of POEGs or their components according to the references reported in this review.

Field of Application	POEGs or Their Components	Application	Reference
Biotechnology	POEG powder	Melanin production by *Streptomyces roseochromogenes*	[8]
	Cellulose component	Cellulase production by *Streptomyces roseochromogenes*	[16]
	POEG fibers	Cellulase and xylanase production by *Microbacterium metallidurans* TL13	[17]
	POEG fibers	Nanofibrillated cellulose production	[18]
	POEGs	Supplementation of the medium composition of brassica microgreens	[19]
	POEG fibers	Medium to boost lentice germination	[20]
Enviromental decontamination	Activated POEG powder	Blackish water decontamination from methylene blue and Pb^2+^	[21]
	POEG fibers	Water decontamination from Pb^2+^, Cu^2+^, Ni^2+^, Zn^2+^ and Cd^2+^	[22]
	Activated POEG powder	Water decontamination from Cr^6+^	[23]
	POEGs and POEG powder	Sea decontamination from hydrocarbon spill pollution	[24]
	POEG powder	Water decontamination from antibiotics like oxytetracycline	[25]
	POEGs	Sea decontamination from plastics	[26]
Bioplastic and biocomposite preparation	Lignin-carbohydrate complex and nanocrystalline cellulose	Reinforcing agents for biodegradable films obtained from hemp	[2,12]
	Lignin-containing cellulose micro/nanofibrils	Packaging materials	[27]
	POEG fibers	Reinforcing agents in matrices of high-density polyethylene and maleated polyethylene	[28,29]
	POEG fibers	Reinforcing agents in matrices of unsaturated polyester resin matrices	[30]
	POEG fibers	Reinforcing agents in polyhydroxybutyrate-co-hydroxyvalerate matrix	[31]
	POEG fibers	Reinforcing agents in polyhydroxybutyrate-co-hydroxyvalerate based bio-containers	[32]
Construction materials	POEGs	Insulating materials in building	[33]
	POEG fibers	Additive in plasters	[34]
	POEG fibers	Additive in cement composites	[35,36]

## Data Availability

Not applicable.
